# Analyses Mutations in *GSN, CST3, TTR*, and *ITM2B* Genes in Chinese Patients With Alzheimer’s Disease

**DOI:** 10.3389/fnagi.2020.581524

**Published:** 2020-09-10

**Authors:** Yaling Jiang, Bin Jiao, Xinxin Liao, Xuewen Xiao, Xixi Liu, Lu Shen

**Affiliations:** ^1^Department of Neurology, Xiangya Hospital, Central South University, Changsha, China; ^2^National Clinical Research Center for Geriatric Disorders, Central South University, Changsha, China; ^3^Key Laboratory of Hunan Province in Neurodegenerative Disorders, Central South University, Changsha, China; ^4^Key Laboratory of Organ Injury, Aging and Regenerative Medicine of Hunan Province, Changsha, China

**Keywords:** Alzheimer’s disease, hereditary amyloidosis, gelsolin, genetics, China

## Abstract

Amyloid protein deposition is a common mechanism of hereditary amyloidosis (HA) and Alzheimer’s disease (AD). Mutations of g*elsolin* (*GSN*), *cystatin C* (*CST3*), *transthyretin* (*TTR*), and *integral membrane protein 2B* (*ITM2B*) genes can lead to HA. But the relationship is unclear between these genes and AD. Genes targeted sequencing (GTS), including *GSN, CST3, TTR*, and *ITM2B*, was performed in a total of 636 patients with clinical AD and 365 normal controls from China. As a result, according to American College of Medical Genetics and Genomics (ACMG) guidelines, two novel likely pathogenic frame-shift mutations (GSN:c.1036delA:p.K346fs and GSN:c.8_35del:p.P3fs) were detected in five patients with AD, whose initial symptom was memory decline, accompanied with psychological and behavioral abnormalities later. Interestingly, the patient with K346fs mutation, presented cerebral β-amyloid protein deposition, had an early onset (48 years) and experienced rapid progression, while the other four patients with P3fs mutation had a late onset [(Mean ± SD): 69.50 ± 5.20 years] and a long course of illness [(Mean ± SD): 9.24 ± 4.86 years]. Besides, we also discovered 17 variants of uncertain significance (VUS) in these four genes. To our knowledge, we are the first to report AD phenotype with *GSN* mutations in patients with AD in the Chinese cohort. Although mutations in the *GSN* gene are rare, it may explain a small portion of clinically diagnosed AD.

## Introduction

Alzheimer’s disease (AD) is a progressive neurodegenerative disease and is the most common form of dementia in the elderly, which mainly characterized by the progressive decline in memory and cognitive function. Epidemiological data showed that there were 50 million AD patients worldwide in 2018, and it was expected to grow to 152 million by 2050 (International AsD, 2019). Although the vast majority of AD occur on a sporadic basis, mutations in three genes [*amyloid precursor protein* (*APP*), *presenilin 1* (*PSEN1*), and *presenilin 2* (*PSEN2*)] could lead to rare familial AD (FAD; <0.5%), whose symptoms occur earlier than sporadic AD, usually between 30 and 50 years of age, also named as early-onset AD. “Typical” late-onset AD may be motivated by a complex interaction between genetic and environmental factors, usually more than 65 years of age. It is currently believed that about 70% of AD risk can be attributed to genetic factors (Bateman et al., 2011; Lane et al., 2018). The prevalent theory of AD pathogenesis is the amyloid hypothesis, suggesting that accumulation of pathological forms of β-amyloid protein (Aβ) is the primary pathological process (Lane et al., 2018).

Hereditary amyloidosis (HA) represents a series of single-gene diseases that caused by amyloidogenic precursor protein genes mutations (Chyra Kufova et al., 2018). There are four genes, which were *gelsolin* (*GSN*), *cystatin C* (*CST3*), *transthyretin* (*TTR*), and *integral membrane protein 2B* (*ITM2B*), whose mutations can lead to autosomal dominant HA, while playing an important role in the pathogenesis of AD (Ray et al., 2000; Sastre et al., 2004; Hirko et al., 2007; Mi et al., 2007; Buxbaum et al., 2008; Buxbaum and Johansson, 2017; Tamayev et al., 2011; Matsuda and Senda, 2019). Established associations between these genes and HA include *GSN* and familial amyloidosis of the Finnish type (FAF; Nikoskinen et al., 2015), *TTR* and transthyretin-related amyloidosis (AMYL-TTR; Sekijima, 2015), *ITM2B* and familial British dementia or familial Danish dementia (Del Campo and Teunissen, 2014), as well as *CST3* and cerebral amyloid angiopathy (Abrahamson et al., 1989). The *GSN* gene encodes gelsolin, which is a calcium-regulated actin regulatory protein that involved in inflammation, cell movement, apoptosis, and cancer development. The gelsolin protein could also inhibit the fibrillization of Aβ, and defibrillize its preformed fibrils (Ray et al., 2000; Hirko et al., 2007). The cystatin C protein is an inhibitor of cysteine proteinases, which could inhibit amyloid fibril formation and Aβ deposition (Sastre et al., 2004; Mi et al., 2007). The transthyretin protein, a thyroid hormone-binding protein, contains a BRICHOS domain, which could serve as the efficient inhibitor of Aβ fibril formation and toxicity (Buxbaum et al., 2008; Buxbaum and Johansson, 2017). Then integral membrane protein 2B, a type II transmembrane protein, could bind APP and inhibit all alpha, beta, and gamma pathways of APP proteolysis (Tamayev et al., 2011; Matsuda and Senda, 2019). In summary, they all could play as physiological inhibitors of Aβ under specific conditions, which might be associated with AD.

Although there are few reports of *CST3* (Hua et al., 2012; Paz-Y-Miño et al., 2015) and *TTR* (Sassi et al., 2016; Xiang et al., 2017) genes in patients with AD, there is no report of *GSN* and *ITM2B* genes. Our study is the first to screen mutations in *GSN*, *CST3*, *TTR*, and *ITM2B* genes in patients with AD by genes targeted sequencing (GTS).

## Materials and Methods

### Subjects

The study included 636 AD patients in China [female 59.3%, onset age (Mean ± SD): 66.17 ± 11.18 years]. Patients were diagnosed by at least two experienced doctors of Xiangya Hospital according to the National Institute of Neurological and Communicative Disorders and Stroke and the Alzheimer’s Diseases and Related Disorders Associations (NINCDS–ADRDA). A total of 365 cognitive normal individuals (MMSE ≥ 27) were recruited from a physical examination center of Xiangya hospital [female 52.1%, age (Mean ± SD): 70.65 ± 5.35 years]. All subjects signed informed consent. Patients carrying with pathogenic genes of AD (*APP, PSEN1, PSEN2*) and vascular cognitive impairment [*notch receptor 3* (*NOTCH3*), *HtrA serine peptidase 1* (*HTRA1*), *collagen type IV alpha* *1 chain* (*COL4A1*), *three prime repair exonuclease 1* (*TREX1*), *galactosidase alpha* (*GLA*)] were excluded.

### Genes Targeted Sequencing and Data Analysis

GTS, including *GSN* (*NM_000177.4*), *CST3* (*NM001288614.1*), *TTR* (*NM000371.3*), and *ITM2B* (*NM_021999.4*), was performed in all subjects. Genomic DNA of all samples was extracted according to the manufacturer’s standard procedure using the QIAamp DNA Blood Midi Kit (Qiagen, Hilden, Germany). Then the genomic DNA was fragmented by Covaris LE220 (MA, USA) to generate paired-end library (200–250 bp) and constructed into the libraries. The baits, a pool of 423 individually synthesized 5′-biotinylated 120 bp RNA oligonucleotides, cover four genes related with Aβ protein processing in HA. The targeted regions were captured with the baits as described below. DNA libraries (1 μg each) were mixed with the adaptor blockers and 5 μg of Cot-I DNA. The DNA mixture was denatured at 95°C for 5 min and then snap cooled on ice immediately. Next, the denatured DNA mixture and baits were transferred into hybridization solution (6× SSC, 1% SDS, 5× Denhardt’s Solution). Hybridization was performed at 65°C for 4 h. After hybridization, the capture chip was washed with 2× SSC and 0.1% SDS for 5 min and 0.2× SSC and 0.1% SDS for 2 × 5 min at 55°C. The captured DNAs were eluted with 100 μl of TE at 95°C for 10 min and purified by using a PCR clean-up kit. The eluted DNAs were subjected to 15 cycles of PCR amplification using the Illumina P5 and P7 primers and subjected to another round of hybridization capture with the same conditions. The products were then subjected to Agilent 2100 Bioanalyzer and ABI StepOne to estimate the magnitude of enrichment. After quality control, captured library sequencing was carried out on Illumina HiSeq X Ten Analyzers (Illumina, San Diego, CA, USA). Following the manufacturer’s standard sequencing protocols for 150 cycles per read to generate paired-end reads. Image analysis, error estimation, and base calling were performed using Illumina Pipeline software to generate raw data.

Then, we performed bioinformatics processing and data analysis to detect the potential variants. We using AfterQC to generate “clean reads” for further analysis. The “clean reads” (with a length of 150 bp) derived from targeted sequencing and filtering were then aligned to the human genome reference (hg19) using the BWA (Burrows Wheeler Aligner) software. After alignment, the output files were used to perform sequencing coverage and depth. We used GATK (Genome Analysis Toolkit) software^1^ to detect SNVs and indels. All SNVs and indels were filtered and estimated *via* multiple databases, including Genome AD (Genome Aggregation Database dataset) and ExAC (The Exome Aggregation Consortium dataset). We used dbNSFP (Liu et al., 2016) to predict the effect of missense variants. Pathogenic variants were assessed by the American College of Medical Genetics and Genomics (ACMG) guidelines (Richards et al., 2015).

### Sanger Sequencing

All likely pathogenic variants were screened using sanger sequencing. The sanger sequencing was amplified using identical forward and reverse primers (GSN-K346fs-F: 5′-CTTCCCATGTGCAGTTTGTGTT-3′, GSN-K346fs-R: 5′-AGCCCAAGACTTCTGATTTCCA-3′; GSN-P3fs-F: 5′-GCCTCGGTGAAAAGCTTTCAAA-3′, GSN-P3fs-R: 5′-TTTCCTAGCGCTGTATCTGCAA-3′). All PCR products were sequenced with Big Dye terminator v3.1 sequencing chemistry on an ABI 3730xl DNA analyzer (Applied Biosystems). DNA sequences were analyzed using sequencing software of Mutation Surveyor (Softgenetics).

### Multiple Sequence Alignment and Structure Modeling

To evaluate the effect of the novel frame-shift mutations on structure and function of proteins, multiple sequence alignment was analyzed by T-Coffee^2^, and three-dimensional (3D) models of the mutant protein structures were built by Discovery Studio software. We used homology models of gelsolin in the Protein Data Bank (PDB) to construct the 3D structure of the mutant proteins by Discovery Studio software.

## Results

This study included 636 patients with clinical AD and 365 cognitive normal controls from China, and the basic information of patients and controls was shown in Table 1. According to the ACMG guidelines, we identified two novel “likely pathogenic” mutations and 11 variants of uncertain significance (VUS) in the *GSN* gene, two VUS in the *CST3* gene, two VUS in the *ITM2B* gene, and two VUS in the *TTR* gene in patients with AD in the Chinese cohort (Table 2). The two novel “likely pathogenic” mutations were not detected in normal controls. The first “likely pathogenic” mutation (PVS1 + PM2) in the *GSN* gene was c.1036delA:p.K346fs, whose frequency in all databases was not available (NA), such as East Asian population of Genome Aggregation Database dataset (gnomAD_genome_EAS), All population of Genome Aggregation Database dataset (gnomAD_genome_ALL), and East Asia population of the Exome Aggregation Consortium (ExAC_EAS). The second “likely pathogenic” mutation (PVS1 + PM2) in the GSN gene was c.8_35del:p.P3fs, whose frequency in gnomAD_genome_EAS was 0.0049, in gnomAD_genome_ALL was 0.0003, and in ExAC_EAS was 0, suggesting that the P3fs mutation was only detected in East Asian populations. The multiple sequence alignment and 3D models of the mutant protein structures were shown in Figure 3.

**Table 1:** Basic information of patients and controls.

	AD patients	Cognitive normal controls
Numbers	636	365
Age of onset (years)	66.17 ± 11.18	70.65 ± 5.35
Gender		
Male	259	175
Female	377	190
Race		
Han nationality	630	365
Others	6	0
APOE		
ε2/ε2	2	2
ε2/ε3	50	54
ε2/ε4	12	6
ε3/ε3	321	237
ε3/ε4	207	63
ε4/ε4	44	3

**Table 2:** Variants in genes of *gelsolin* (*GSN*), *cystatin C* (*CST3*), *transthyretin* (*TTR*), and *integral membrane protein 2B* (*ITM2B*).

Gene name	Mutation name	ACMG	Patients	Normal controls	Mutation mode	HET/HOM	Risk dbSNP	gnomAD_g enome_EAS	ExAC_EAS	Polyphen2	MutTaster	PROVEAN
*GSN*	c.8_35del:p.P3fs	Likely pathogenic (PVS1 + PM2)	**4**	**0**	Frameshift deletion	HET	rs764841269	4.90E-03	0	NA	NA	NA
*GSN*	c.1036delA:p.K346fs	Likely pathogenic (PVS1 + PM2)	**1**	**0**	Frameshift deletion	HET	NA	NA	NA	NA	NA	NA
*GSN*	c.425G>A:p.R142Q	Uncertain significance (N)	**1**	**0**	Nonsynonymous SNV	HET	rs138153246	0	0	B	D	N
*GSN*	c.613G>A:p.V205M	Uncertain significance (PM2)	**1**	**0**	Nonsynonymous SNV	HET	NA	NA	NA	D	D	N
*GSN*	c.863C >T:p.A288V	Uncertain significance (N)	**3**	**0**	Nonsynonymous SNV	HET	rs780252276	4.06E-04	0.0006	B	N	N
*GSN*	c.902C >T:p.Y301C	Uncertain significance (PM2)	**2**	**0**	Nonsynonymous SNV	HET	rs758752620	5.80E-05	0.0001	D	D	D
*GSN*	c.958C >T:p.P320S	Uncertain significance (N)	**1**	**0**	Nonsynonymous SNV	HET	rs768184900	0	0	D	D	D
*GSN*	c.1055C >T:p.T352M	Uncertain significance (PM2 + BP4)	**1**	**0**	Nonsynonymous SNV	HET	NA	NA	NA	B	N	N
*GSN*	c.1406C >T:p.Y469C	Uncertain significance (PM2)	**1**	**1**	Nonsynonymous SNV	HET	rs375227932	4.06E-04	0.0003	D	D	D
*GSN*	c.1655dupC:p.S522fs	Uncertain significance (PM4)	**1**	**0**	Frameshift insertion	HET	rs769989772	1.76E-04	0.0003	NA	NA	NA
*GSN*	c.1730G >T:p.R577L	Uncertain significance (PM2)	**1**	**0**	Nonsynonymous SNV	HET	rs528604896	1.11E-03	0.001	D	D	D
*GSN*	c.1793C >T:p.T598I	Uncertain significance (N)	**2**	**1**	Nonsynonymous SNV	HET	rs376326631	1.16E-04	0.0001	D	D	D
*GSN*	c.2198C >T:p.T733M	Uncertain significance (PM2)	**1**	**0**	Nonsynonymous SNV	HET	rs142854368	0	0	D	D	D
*CST3*	c.236G >T:p.R79L	Uncertain significance (PM2 + BP4)	1	0	Nonsynonymous SNV	HET	NA	NA	NA	P	N	D
*CST3*	c.371C >T:p.S124F	Uncertain significance (PM2)	2	0	Nonsynonymous SNV	HET	rs754306266	0	0	D	D	D
*TTR*	c.62G>C:p.G21A	Uncertain significance (PM2)	1	0	Nonsynonymous SNV	HET	NA	NA	NA	B	N	N
*TTR*	c.370C >T:p.R124C	Uncertain significance (PM2 + PP3)	1	1	Nonsynonymous SNV	HET	rs745834030	4.64E-04	0.0001	P	N	N
*ITM2B*	c.20C >T:p.N7S	Uncertain significance (N)	1	2	Nonsynonymous SNV	HET	rs779234032	0	0	B	D	N
*ITM2B*	c.325G >T:p.A109S	Uncertain significance (N)	2	2	Nonsynonymous SNV	HET	rs748146945	5.22E-04	0.0003	B	D	N

*PVS1: predicted null variant in a gene where loss of function (LOF) is a known mechanism of disease. PM2: absent in population databases. PM4: protein length changing variant. PP3: multiple lines of computational evidence support a deleterious effect on the gene/gene product. BP4: multiple lines of computational evidence suggest no impact on gene/gene product. Uncertain significance (N): does not meet any ACMG standards. Polyphen2 (D: probably damaging, P: possibly damaging; B: benign). MutTaster (D: disease causing; N: polymorphism). Provean (D: deleterious SNV; N: neutral). gnomAD_genomeEAS: frequency in the East Asian population of Genome Aggregation Database dataset; ExACEAS: frequency in East Asia population of the Exome Aggregation Consortium (ExAC) database; HET: heterozygous; HOM: homozygous; NA: not available; SNV: single nucleotide variant*.

**Figure 1:**
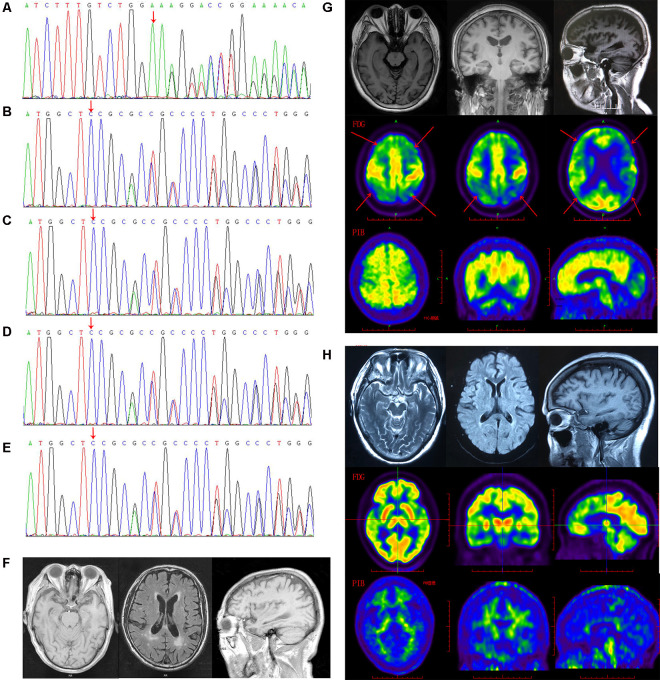
The *gelsolin* (*GSN*) gene mutation sequencing diagram and patients’ imaging. **(A)** The sequencing diagram of case 1 (GSN:c.1036delA:p.K346). **(B)** The sequencing diagram of case 2 (GSN:c.8_35del:p.P3fs). **(C)** The sequencing diagram of case 3 (GSN:c.8_35del:p.P3fs). **(D)** The sequencing diagram of case 4 (GSN:c.8_35del:p.P3fs). **(E)** The sequencing diagram of case 5 (GSN:c.8_35del:p.P3fs). **(F)** The magnetic resonance imaging (MRI) of case 4 with P3fs mutation. **(G)** The MRI, FDG-PET and PIB-PET of case 1 with K346fs mutation. **(H)** The MRI, FDG-PET and PIB-PET of case 5 with P3fs mutation).

**Figure 2:**
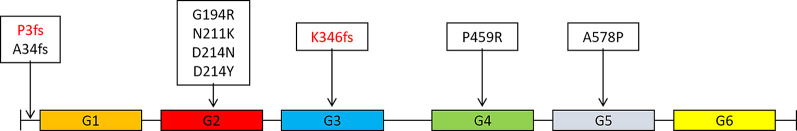
Distribution of mutations in the *GSN* gene in different domains of gelsolin protein (Gelsolin protein has six domains, named G1 to G6).

**Figure 3:**
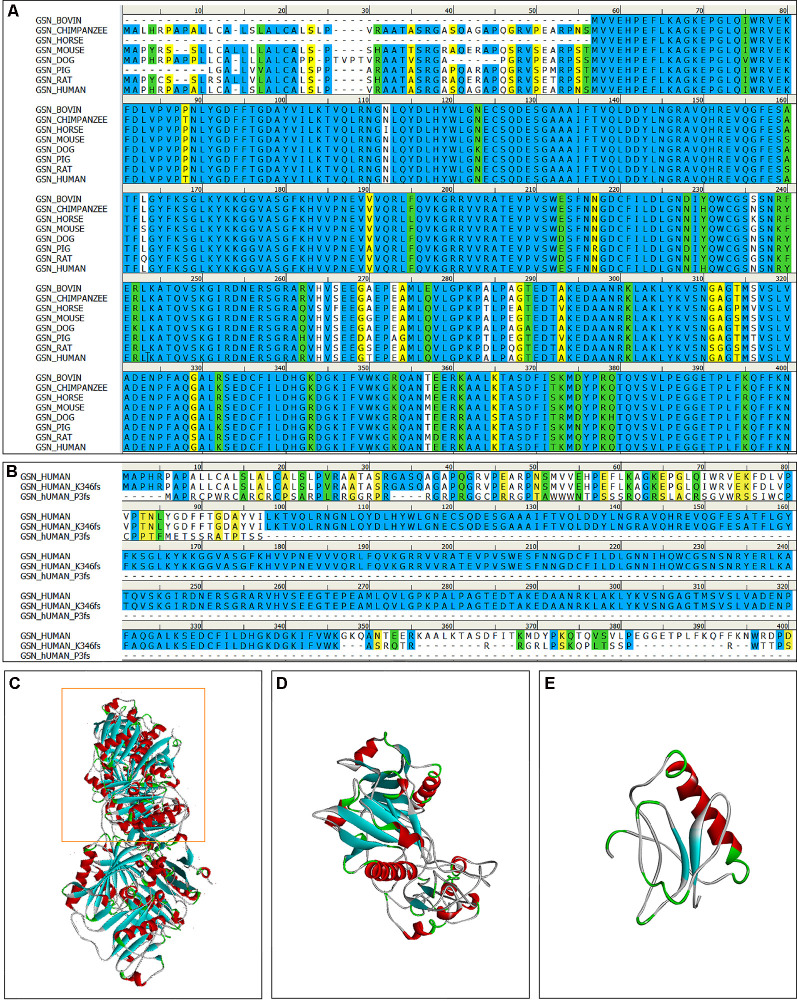
The multiple sequence alignment and 3D models of the mutant protein structures. **(A)** The multiple sequence alignment of the gelsolin protein among vertebrates (amino acid residues 1–400); The degree of conservation between sequences was showed in different colors (identical: bule, strong: green, weak: yellow, non-matching: white). **(B)** The multiple sequence alignment of the gelsolin protein, K346fs and P3fs mutant gelsolin protein (amino acid residues 1–400). **(C)** 3D model of the wild-type gelsolin. **(D)** 3D model of the mutant protein with K346fs mutation, which is similar to part of the wild-type gelsolin (yellow box in **C**). **(E)** 3D model of the mutant protein with P3fs mutation.

The K346fs mutation was detected in a sporadic female patient whose onset age was 48 years old (case 1; Figure 1A). The patient first presented memory decline, manifested as forgetting things just done. Simultaneously, she became apathetic and did not communicate with others. About 1 year later, she could not recognize her relatives or take care of herself in daily life such as wearing clothes and bathing. She also developed psychiatric symptoms about the same time, which were emotionally violent, hitting people and crying for no reasons. Cognitive assessments: (1) mini-mental state examination (MMSE): 9/30; (2) montreal cognitive assessment scale (MoCA): 2/30; (3) neuropsychiatric inventory (NPI): 6; (4) daily living ability scale (ADL): 37; and (5) clinical dementia rating scale (CDR): 2. Electroencephalogram (EEG) showed moderate abnormal EEG (frontal and temporal regions were paroxysmal slow waves). Brain magnetic resonance imaging (MRI) showed mild leukoencephalopathy and brain atrophy (Figure 1G). Positron emission tomography-computed tomography (PET-CT) revealed: (1) fluorodeoxyglucose (FDG) metabolism decreased in bilateral frontal and parietal lobes; (2) diffuse Aβ protein deposition in bilateral cerebral cortex and subcortical nuclei; (3) lacunar infarction in brain stem; and (4) brain atrophy (Figure 1G). In addition, ophthalmologic symptoms are common in FAF patients. The ophthalmologic examinations of the case 1 patient revealed that the cornea was normal, but bilateral optic nerves were atrophied. Unfortunately, the patient refused to do the skin biopsy.

The P3fs mutation was detected in four sporadic patients (case 2–5; Table 3), whose onset age was older than 65 years [onset age (Mean ± SD): 69.50 ± 5.20 years; Figures 1B–E). The initial symptom was memory decline and the symptom progressed slowly in all patients. Among them, case 2, case 3, and case 4 patients experienced cognitive disturbance (spatial disorientation and count disturbance) and behavior change (became irritable and prone to temper) later. The case 2 patient developed behavioral and psychological symptoms in the third year after onset. The case 5 patient, who had a shorter course, did not suffer above symptoms, and her clinical manifestations were milder than other patients. Case 2 and case 3 patients cannot be traced at present. The case 4 patients were seriously ill and stayed in bed all day during our follow-up, so he could not cooperate with our study. The symptom of case 5 patient has not changed obviously so far, and she did more examinations during the follow-up. PET-CT revealed: (1) FDG imaging showed no abnormal increase or decrease in glucose metabolism in the brain; (2) PIB imaging showed no abnormality of imaging agent uptake in cerebral cortex, and suggested no obvious Aβ protein deposition in the cerebral cortex; and (3) mild brain atrophy (Figure 1H). As the case 5 patient had a history of numbness of the limbs, an extra electromyogram (EMG) was performed. Nerve conduction velocity (NCV) was normal in bilateral median nerve, ulnar nerve, tibial nerve, common peroneal nerve, and sural nerve. EMG showed no significant changes were observed in the limb muscles and no obvious abnormality in the skin sympathetic response of the extremities. Ophthalmic examination showed that the cornea was normal. Unfortunately, the patient refused to do the skin biopsy.

**Table 3:** Information of five patients with mutations in the *GSN* gene.

	Case 1	Case 2	Case 3	Case 4	Case 5
Mutation	K346fs	P3fs	P3fs	P3fs	P3fs
Gender	Female	Female	Male	Male	Female
Onset age	48	77	69	66	66
Visiting ages	50	80	75	68	67
Course (years)	4	12	14	8	3
First symptoms	Memory decline	Memory decline	Memory decline	Memory decline	Memory decline
Additional symptoms	Behavioral and Psychological symptoms	Behavioral and Psychological symptoms	Behavior change	Behavior change	No
Past medical history		Coronary heart disease	Cerebral infarction, hypertension, rhinitis, left inguinal hernia	Hypertension, hyperlipidemia, diabetes	Headache, numbness of the limbs, patent foramen ovale
Family history	No	No	No	No	No
APOE	ε3/ε4	ε3/ε3	ε3/ε3	ε3/ε3	ε3/ε4
Cognitive Assessment
MMSE	9/30	9/30	0/30	18/30	23/30
MoCA	2/30	8/30	0/30	14/30	15/30
ADL	37	61	-	-	23
NPI	6	-	-	-	7
CDR	2	-	-	-	0.5
MRI	Mild leukoencephalopathy and brain atrophy (Figure 1G)	-	-	Multiple lacunar infarction in the brain, leukoencephalopathy, brain atrophy (Figure 1F)	Mild brain atrophy and mild leukoencephalopathy (Figure 1H)

## Discussion

In our study, we screened mutations of *GSN, CST3, TTR*, and *ITM2B* genes by GTS in patients with AD in China, and identified two novel “likely pathogenic” mutations K346fs and P3fs in the *GSN* gene, suggesting that *GSN* gene may explain a small portion of AD.

The *GSN* gene is located on the chromosome 9q33.2 and is inherited by dominance. Up to now, seven pathogenic mutations in the *GSN* gene have been reported in worldwide, namely A34fs, G194R, N211K, D214N, D214Y, P459R, and A578P (Figure 2 and Table 4, Hiltunen et al., 1991; Stewart et al., 2000; Conceição et al., 2003; Chastan et al., 2006; Ardalan et al., 2007; Huerva et al., 2007; Carrwik and Stenevi, 2009; Luttmann et al., 2010; Makioka et al., 2010; Asahina et al., 2011; Solari et al., 2011; Taira et al., 2012; Sethi et al., 2013; Efebera et al., 2014; Park et al., 2016; Caress et al., 2017; de Souza et al., 2017; Feng et al., 2018; Mustonen et al., 2018; Oregel et al., 2018; Sridharan et al., 2018). The D214N/Y mutation is the most common mutation and could cause the disease of FAF, which mainly manifested as corneal lattice dystrophy, cranial neuropathy, peripheral neuropathy, and cutis laxa (Nikoskinen et al., 2015). FAF has also been reported in other areas besides Finland. Due to differences in regions and races, it could be seen that, in East Asia, the clinical manifestations of FAF were mainly neurological symptoms (Taira et al., 2012; Park et al., 2016; Feng et al., 2018). Followed by G194R and N211K mutations, whose clinical phenotype is different from D214N/Y, mainly gelsolin-related renal amyloidosis (Sethi et al., 2013; Efebera et al., 2014). A34fs, P459R, and A578P mutations were reported recently, corresponding totally different manifestations from mutations that we mentioned before (Feng et al., 2018; Oregel et al., 2018; Sridharan et al., 2018; Table 4). Patients with A34fs mutation presented with seizures and brain lesions, without skin and eye symptoms. The patient with P459R mutation manifested as cranial nerve palsy (facial nerve) and proximal muscle weakness, then dead due to unexplained dyspnea and severe sepsis. The patient with A578P mutation combination with V122I mutation in the TTR gene (mainly related to cardiac involvement), characterized by progressive dyspnea, without cranial nerve, eye, and skin symptoms. That is to say, the *GSN* gene has a heterogeneity between genetic phenotype and clinical phenotype, different mutations lead to different locations of the lesions, resulting in different clinical manifestations. But there was no report of the AD phenotype, and we are the first to report that K346fs and P3fs mutations in the *GSN* gene may lead to AD.

**Table 4:** Pathogenic mutations in the *GSN* gene in worldwide.

Mutation	Area	Disease	Pathogenic protein deposition	Clinical manifestations
P3fs*	China	AD	Not known, maybe brain	Cognitive dysfunction, mild peripheral neurological symptoms, no eye or skin symptoms.
A34fs	China (Feng et al., 2018)	Atypical FAF	Not known, maybe brain and cerebral vessels.	Seizures and brain lesions. no skin, or eye symptoms.
G194R	USA (Sethi et al., 2013)	Gelsolin-related renal amyloidosis	Kidney	Chronic kidney disease and anemia.
N211K	USA (Efebera et al., 2014)	Gelsolin-related renal amyloidosis	Kidney	Nephrotic range proteinuria of 13.2 g/day as the only presenting symptom.
D214N/Y	Finland (Hiltunen et al., 1991; Mustonen et al., 2018), USA (Caress et al., 2017), Japan (Makioka et al., 2010; Asahina et al., 2011; Taira et al., 2012), Spain (Huerva et al., 2007), France (Chastan et al., 2006), Portugal (Conceição et al., 2003), England (Stewart et al., 2000), Iran (Ardalan et al., 2007), Brazil (Solari et al., 2011; de Souza et al., 2017), Sweden (Carrwik and Stenevi, 2009), Germany (Luttmann et al., 2010), Korea (Park et al., 2016)	FAF	Eye, nerve, and skin	The main clinical manifestations are corneal lattice dystrophy, cranial neuropathy, peripheral neuropathy and cutis laxa. in East Asia (Japan and Korea), the clinical manifestations of FAF were mainly neurological symptoms.
K346fs*	China	AD	Not known, maybe brain	Cognitive dysfunction, personality changes, psychiatric symptoms, symptoms in multiple systems of the body (eyes, skin and thyroid).
P459R	USA (African descent; Oregel et al., 2018)	Atypical FAF	Muscle tissue	Cranial nerve palsy (facial nerve) and proximal muscle weakness, then dead due to unexplained dyspnea and severe sepsis. The MRI of the head and spinal cord was normal. Biopsy of left quadriceps femoris biopsy showed focal myopathy and denervation atrophy (severe, type II).
A578P	USA (Sridharan et al., 2018)	ATTR (transthyretin amyloidosis)	Myocardium (amyloid deposition); abdominal fat and rectum mucosa (gelsolin deposition).	Combination with V122I mutation of the *TTR* gene (mainly related to cardiac involvement), characterized by progressive dyspnea, no cranial nerve, eye or skin symptoms.

*Novel mutation.

Gelsolin protein consists six domains, named G1 to G6. Most mutations currently found (D214N/Y, G194R, and N211K) were located in the G2 domain (Figure 2), affecting the stability of the G2 domain and leading to disease (Bonì et al., 2016, 2018; Giorgino et al., 2019). Recently, Zorgati et al. (2019) proposed a new hypothesis that the D214N/Y mutation affected the stability of the G2 domain by affecting the interactions between G2–G3 domains. They validated this hypothesis by making G3 domain non-FAF mutations (K341M, L388D, and Q391L), confirming that these mutations disrupted the interactions of G2 and G3 domains, making the cleavage site more susceptible to exposure; and they also predicted that mutations in the G3 domain will also lead to disease. Our newly identified K346fs mutation was located in the G3 domain, which was near the sites that we mentioned above, and might promote the occurrence of disease in the similar way. Moreover, the case 1 patient carried K346fs mutation started disease early, and her PIB-PET showed Aβ deposition. Therefore, we considered that the K346fs mutation is most likely to be pathogenic.

Both the P3fs mutation, which was our newly identified, and the A34fs mutation had a frame shift at the start site. Due to the frame shift at the start site, some scholars believed that it might not translate the functional domain of gelsolin. Therefore, the amyloid protein formed by the A34fs mutation might have different composition relative to other FAF fibrils (Feng et al., 2018; Zorgati et al., 2019). Both patients with A34fs and P3fs mutations mainly presented with central nervous symptoms, without skin, eyes, and peripheral nervous symptoms. All patients (case 2–5) with P3fs mutation had a late onset of disease, and the PIB-PET showed no Aβ deposition in the case 5 patient. But the patient with A34fs mutation reported recently did not have a biopsy of skin or other sites. It was uncertain whether the patient had the deposition of gelsolin protein. Although the P3fs mutation was assessed as “likely pathogenic” by ACMG, we considered that it needed more studies to verify.

There was also a close relationship between AD and gelsolin protein. The level of gelsolin changed as AD progressed (Antequera et al., 2009; Guntert et al., 2010; Peng et al., 2015; Yao et al., 2018). Mechanism studies found that gelsolin contained two Aβ binding sites (Chauhan et al., 1999), through binding to Aβ protein, gelsolin could inhibit Aβ-induced toxicity (Harms et al., 2004; Qiao et al., 2005), inhibit Aβ fibrosis and degrade fibers that already formed (Ray et al., 2000; Hirko et al., 2007). Studies also showed that injection or over expression of gelsolin resulted in a significant reduction in amyloid loads and a decrease in Aβ levels in AD transgenic mice (Hirko et al., 2007; Antequera et al., 2009; Yang et al., 2014). In general, gelsolin acted as an anti-amyloid-forming protein and had neuroprotective effects in AD patients. When the *GSN* gene mutated in AD patients, its protective effect on the nerve might be decreased, thus promoting the occurrence of AD.

Some variants in genes of *CST3*, *IMT2B*, and *TTR* were detected in our study, but they were not pathogenic. Although some evidence suggested that the cystatin C could affect the Aβ protein processing (Kaur and Levy, 2012), there were no pathogenic mutations found in the *CST3* gene in Chinese patients with AD (Hua et al., 2012; Paz-Y-Miño et al., 2015), which was similar to our results. As for the *TTR* gene, some potential pathogenic mutations were reported in patients with AD (Sassi et al., 2016; Xiang et al., 2017), which was different to our results. The reasons for the difference might be our smaller sample size and different races. The *ITM2B* gene was similar to *CST3* (Fotinopoulou et al., 2005; Matsuda et al., 2005), and no related pathogenic mutations were reported. Therefore, we suspected that these three genes may not be closely related to AD.

In summary, we are the first to report AD phenotype with *GSN* mutation in patients with AD in Chinese cohort, expanding the *GSN* gene mutation spectrum and its corresponding clinical phenotype spectrum. Although mutations in the *GSN* gene are rare, it may explain a small portion of AD.

## Data Availability Statement

The datasets presented in this study can be found in online repositories. The names of the repository/repositories and accession number(s) can be found below: BioProject NCBI, accession no.: PRJNA656640 (https://www.ncbi.nlm.nih.gov/bioproject/PRJNA656640).

## Ethics Statement

The studies involving human participants were reviewed and approved by Ethics Committee of National Center for Geriatrics Clinical Medical Research, China. The patients/participants provided their written informed consent to participate in this study. Written informed consent was obtained from the individual(s) for the publication of any potentially identifiable images or data included in this article.

## Author Contributions

LS designed the experiment. YJ performed the experiment. YJ, XX and XLiu processed the data. YJ wrote the article. BJ and XLiao modified the article.

## Conflict of Interest

The authors declare that the research was conducted in the absence of any commercial or financial relationships that could be construed as a potential conflict of interest.

## Footnotes

^1^https://software.broadinstitute.org/gatk

^2^https://www.ebi.ac.uk/Tools/msa/tcoffee/
